# Ultrafast and ultralarge multiple sequence alignments using TWILIGHT

**DOI:** 10.1093/bioinformatics/btaf212

**Published:** 2025-07-15

**Authors:** Yu-Hsiang Tseng, Sumit Walia, Yatish Turakhia

**Affiliations:** Department of Electrical and Computer Engineering, University of California San Diego, San Diego, CA 92093, United States; Department of Electrical and Computer Engineering, University of California San Diego, San Diego, CA 92093, United States; Department of Electrical and Computer Engineering, University of California San Diego, San Diego, CA 92093, United States

## Abstract

**Motivation:**

Multiple sequence alignment (MSA) is a fundamental operation in bioinformatics, yet existing MSA tools are struggling to keep up with the speed and volume of incoming data. This is because the runtimes and memory requirements of current MSA tools become untenable when processing large numbers of long input sequences, and they also fail to fully harness the parallelism provided by modern CPUs and GPUs.

**Results:**

We present **T**all and **Wi**de A**lig**nments at **H**igh **T**hroughput (**TWILIGHT**), a novel MSA tool optimized for speed, accuracy, scalability, and memory constraints, with both CPU and GPU support. TWILIGHT incorporates innovative parallelization and memory-efficiency strategies that enable it to build ultralarge alignments at high speed even on memory-constrained devices. On challenging datasets, TWILIGHT outperformed all other tools in speed and accuracy. It scaled beyond the limits of existing tools and performed an alignment of 1 million RNASim sequences within 30 min while utilizing <16 GB of memory. TWILIGHT is the first tool to align over 8 million publicly available SARS-CoV-2 sequences, setting a new standard for large-scale genomic alignment and data analysis.

**Availability and implementation:**

TWILIGHT’s code is freely available under the MIT license at https://github.com/TurakhiaLab/TWILIGHT. The test datasets and experimental results, including our alignment of 8 million SARS-CoV-2 sequences, are available at https://zenodo.org/records/14722035.

## 1 Introduction

Multiple sequence alignment (MSA) is a powerful tool in bioinformatics for comparing biological sequences with numerous applications, including assembling genomes (Vaser *et al.* 2017), identifying sequence homology (Ali *et al.* 2019), inferring evolutionary trees (Kapli *et al.* 2020), reconstructing ancestral genomes (Vialle *et al.* 2018), and identifying the functional regions of the genome (Hu *et al.* 2021). Additionally, MSA can serve as valuable training data for bioinformatics-related machine learning models, such as for predicting protein structures using AlphaFold (Jumper *et al.* 2021).

Progressive alignment is among the most widely used algorithms for constructing MSAs, as first implemented in tools like ClustalW (Thompson *et al.* 1994a) and MAFFT (Katoh *et al.* 2002). In this approach, sequences are aligned sequentially based on their positions in a guide tree, progressing from closely to more distantly related sequences. However, the quality of the progressive alignment depends on the accuracy of the guide tree. To address this, an iterative approach was introduced in MUSCLE (Edgar 2004), which refines the accuracy of both the guide tree and the MSA over multiple iterations.

As progressive and iterative aligners faced scalability challenges, divide-and-conquer algorithms were introduced in the 2010s in tools like SATé-II (Liu *et al.* 2012) and PASTA (Mirarab *et al.* 2015). These methods divide the guide tree into smaller subtrees, align each subtree individually, and then merge the results. PASTA uses the transitivity merger strategy to accelerate the process and improve accuracy. More recently, tools like MAGUS (Smirnov and Warnow 2021) have further enhanced MSA accuracy for sequences with high evolutionary rates by using graph clustering algorithms.

Despite this progress, MSA tools have struggled to meet the computational demands of the rapidly growing genomic datasets. For example, during the COVID-19 pandemic, millions of SARS-CoV-2 genomes were sequenced globally (Maxmen 2021), yet, despite the need, no available MSA tool could produce an MSA comprising all available sequences. This is because existing tools are unable to simultaneously handle *tall* (many sequences) and *wide* (long sequences) alignments within reasonable runtime and memory constraints. Moreover, modern computers, such as CPUs and GPUs, provide highly parallel architectures, but current MSA tools do not provide GPU support and are also unable to effectively exploit the parallelism available on CPUs.

To address these challenges, we present **T**all and **Wi**de A**lig**nments at **H**igh **T**hroughput (**TWILIGHT**), an innovative MSA tool designed to leverage massive parallelism for efficient handling of tall and wide alignment tasks. TWILIGHT adopts the progressive alignment algorithm and uses recently developed tiling strategies (Turakhia *et al.* 2018, Walia *et al.* 2024) to band alignments with constant traceback memory requirements while maintaining high accuracy. Combined with a divide-and-conquer technique, a novel heuristic dealing with gappy columns, and support for GPU acceleration, TWILIGHT demonstrates exceptional speed and memory efficiency; it can align 1 million RNASim (Guo *et al.* 2009) sequences in under 30 min using <16 GB of memory, thus enabling ultralarge alignments to be performed even on memory-constrained platforms, like laptops and personal servers. For long sequences, it can align 10 000 sequences with a length of 1 million bases in 3.5 h and 8 million SARS-CoV-2 sequences in just 28 h.

## 2 Materials and methods

### 2.1 TWILIGHT overview

**Figure 1.**
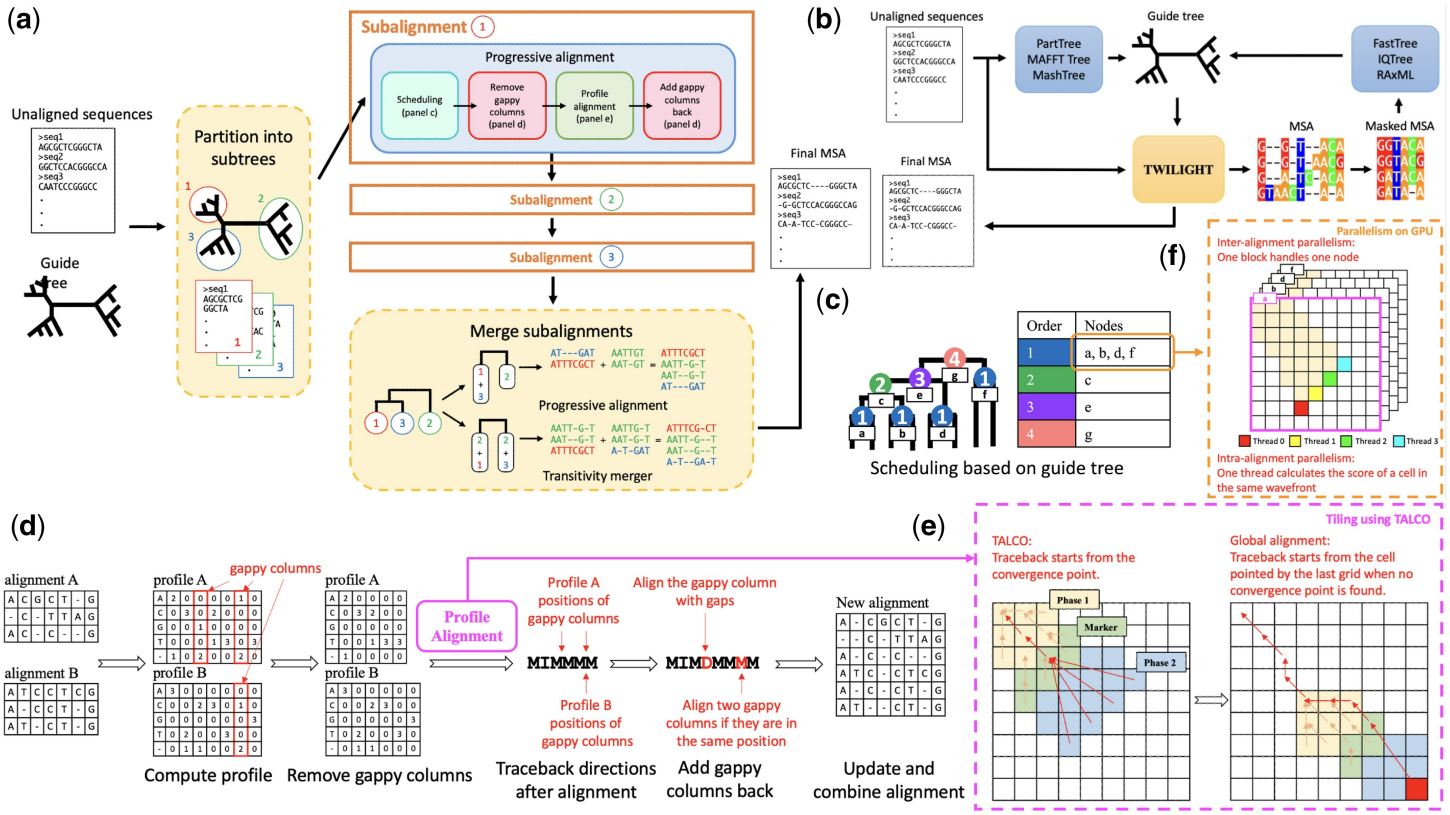
Overview of the TWILIGHT algorithm and its main features. (a) TWILIGHT in its default mode. The progressive alignment is composed of four steps: scheduling, removing gappy columns, profile alignment, and adding gappy columns back. (b) The iterative mode in TWILIGHT. (c) Scheduling alignments. (d) Removing gappy columns and adding them back after profile alignment. (e) Tilling profile alignment using the TALCO algorithm. (f) TWILIGHT exploits two levels of parallelism (inter- and intra-alignment) for profile alignment on the GPU.

TWILIGHT accepts unaligned sequences in FASTA format and an optional input guide tree in Newick format to generate output alignments in FASTA format (Fig. 1a). When a guide tree is unavailable, users can utilize TWILIGHT’s iterative mode (Fig. 1b). In this mode, TWILIGHT estimates an initial guide tree using MashTree (Katz *et al.* 2019), PartTree (Katoh and Toh 2007), or MAFFT’s (Katoh and Standley 2013) default tree generation algorithm (which we refer to as “MAFFT Tree”) for the first iteration. For subsequent iterations, the guide tree is refined based on the alignment inferred from the previous iteration, utilizing a user-specified tree inference tool such as RAxML (Stamatakis 2006), IQ-Tree (Minh *et al.* 2020), or FastTree (Price *et al.* 2010) (see Section 2.3.5).

Within each iteration, TWILIGHT uses a progressive alignment strategy inspired by the extensive body of research in this area (Feng and Doolittle 1987) (Fig. 1a). This strategy builds a MSA step-by-step by first aligning the most similar sequences or sequence groups and then progressively adding less similar sequences or groups based on a guide tree (Fig. 1c). Guide tree construction can be based on a variety of approaches; one common strategy is to use neighbor joining (Saitou and Nei 1987), which takes a distance matrix as input and iteratively grouping sequences with the smallest pairwise distances. Similar to earlier methods (Thompson *et al.* 1994a; Notredame *et al.* 2000, Katoh *et al.* 2002), TWILIGHT aligns sequence groups using their *profiles* (Gribskov *et al.* 1987), which encode the positional frequencies of each residue within the group’s alignment. However, in order to efficiently scale to ultralarge datasets, TWILIGHT introduces novel algorithmic and implementation strategies that address the speed and memory limitations of earlier tools. Specifically, TWILIGHT: (i) is optimized to fully exploit the abundant *parallelism* offered by modern CPU and GPU architectures, and (ii) incorporates sophisticated *memory-efficiency techniques*, enabling it to perform ultralarge alignments within the stringent memory constraints of CPU and GPU devices. Furthermore, TWILIGHT features a novel heuristic of removing gappy columns, allowing it to achieve alignment accuracy that matches or exceeds that of existing multiple-sequence alignment tools. These strategies are detailed further in a later section (see Section 2.3).

### 2.2 Algorithmic details

We now provide a detailed explanation of the individual steps in TWILIGHT, which are also illustrated in Fig. 1:

1. *Divide the guide tree into subtrees:* This is an optional step in TWILIGHT (Fig. 1a), specifically designed for scenarios in which the number of input sequences is so large that they would fit on disk but not within the CPU’s main memory. For such scenarios, TWILIGHT uses a divide-and-conquer algorithm to reduce the CPU’s main memory usage. Specifically, TWILIGHT divides the initial guide tree into smaller subtrees with at most *m* leaves using the centroid decomposition technique defined in SATé-II (Liu *et al.* 2012). The value of *m* is set by the user using a command-line parameter--max-subtree(-m), which is used to ensure that only *m* sequences are loaded by TWILIGHT into the CPU’s main memory at once. This parameter could be set by the user based on available CPU memory and the length of input sequences. Through these optimizations, it is even possible to perform MSA for 1 million SARS-CoV-2 sequences on a laptop device using TWILIGHT. Notably, the default value for--max-subtree is ∞, meaning TWILIGHT performs progressive alignment based on the input guide tree when not specified. Unlike PASTA (Mirarab *et al.* 2015), which utilizes a similar divide-and-conquer approach to enhance speed and accuracy, TWILIGHT primarily uses subtrees to reduce its memory usage, achieving a more efficient computational footprint with a minor trade-off in runtime and accuracy (see Section 4.4).
2. *Progressive alignment of each subtree:* Next, TWILIGHT sequentially iterates over each subtree, loading its corresponding sequences into memory, and computing its *subalignment* using the following four steps:
  - *Scheduling for inter-alignment parallelism:* This step in TWILIGHT identifies which pairs of *profile alignments*—alignments between groups of sequences, where each group may contain one sequence or multiple aligned sequences (Fig. 1d)—that can be performed in parallel based on the structure of the guide tree. This form of parallelism is referred to as *inter-alignment* parallelism. The scheduling algorithm initializes the order of the leaf nodes to 0 and then performs a post-order traversal of the tree (Fig. 1c). Each internal node’s order is set to one greater than the maximum alignment order of its child nodes. Since TWILIGHT only performs pairwise alignments of profiles (Step 2C below), any polytomous node is randomly resolved into a binary tree for processing. In the example illustrated in Fig. 1c, the child nodes of a, b, d, and f can be aligned in parallel during the first step. The remaining nodes, i.e. c, e, and g, have to be processed sequentially and in that order. This approach ensures efficient use of parallel processing resources while respecting the dependencies defined by the guide tree.
  - *Removing gappy columns:* TWILIGHT uses *profiles* to align two groups of aligned sequences based on the progressive strategy. Each profile is a 2-D matrix that records the frequency of each character—the four nucleotides, ambiguous characters (N), and gaps—at every column of its aligned group (Fig. 1d). Therefore, a profile can be easily derived from its alignment. TWILIGHT computes profiles using the same approach as ClustalW (Thompson *et al.* 1994a). However, as more sequences are added, profiles tend to grow longer, often with many columns dominated by gaps. These lengthy profiles can significantly slow down the pairwise profile alignment step (Step 2C), particularly as we approach the root of the guide tree. To address this issue, TWILIGHT identifies and excludes gappy columns—those where gaps constitutes more than a specific threshold (default: 95%)—from the profile alignment step. For instance, in the example in Fig. 1d, using a 60% threshold, three columns with red borders—two in profile A and one in profile B—are identified as gappy columns and removed.
  - *Pairwise profile alignments:* TWILIGHT extends the Needleman–Wunsch algorithm (Needleman and Wunsch 1970) with an affine gap penalty (Gotoh 1982) for pairwise profile alignment, similar to the sum-of-pairs scoring in ClustalW (Thompson *et al.* 1994a). Briefly, this method scores the alignment by calculating the sum of substitution matrix scores for all possible residue pairs in each column of the alignment (see Section 2.3.1). TWILIGHT also provides support for position-specific gap penalty that is used in ClustalW (Thompson *et al.* 1994a), which helps with more gappy alignments as it tends to create more gaps at position where they already exist. Additionally, for enhanced computational efficiency, TWILIGHT uses an adaptive banding strategy, similar to X-Drop (Zhang *et al.* 2000). Since performing pairwise profile alignments is typically the most compute-intensive step of TWILIGHT’s algorithm, it provides GPU acceleration support to optimize its execution. In this case, the CPU computes the profiles and position-specific gap penalties of multiple pairs of sequence groups that can be parallelly aligned (based on the schedule in Step 2A), and sends them to the GPU to perform the profile alignments (see Section 2.3.3).
  - *Adding back gappy columns:* Next, TWILIGHT restores the gappy columns excluded in Step 2B. If gappy columns from both profiles align at the same position, they are merged, reducing the overall alignment length. Otherwise, a gappy column from one profile is aligned with a new gap introduced in the other profile. Empirical evaluations show that this optimization significantly reduces alignment length compared to previous approaches, such as transitivity merger (Mirarab *et al.* 2015), with minimal impact on alignment quality.
  - *Output subalignments to a file:* The earlier step provides the complete MSA of the sequences in the subtree, referred to as *subalignment*, which is then written to an output file in FASTA format. If the initial guide tree was not subdivided into subtrees in Step 1, this file is the final output MSA.
3. *Merging subtree alignments:* At the end of Step 2, each subtree has been individually processed, with its subalignment saved in a separate file. TWILIGHT then follows the guide tree to progressively align these subalignments into the final MSA (Fig. 1a). The size of a profile depends on the alignment length rather than the number of sequences it represents, allowing all profiles to be stored in memory and avoid any high overhead read/write operations in the merging step. These profiles are aligned using either the pairwise profile alignment strategy from Step 2C (default) or the transitivity merger algorithm of PASTA. After each pairwise alignment, the profiles are updated, and the positions for inserting gaps are recorded (see Section 2.3.4). Once the root of the guide tree is reached, TWILIGHT reads the stored subalignment file and adjusts the sequences in each subalignment by inserting gaps at recorded positions. This ensures all subalignments are consistently aligned, with sequences having aligned strings of equal length. The adjusted subalignments are saved to individual files and finally concatenated into a single output MSA in FASTA format using the Unix *cat* utility (Pike and Kernighan 1984).

### 2.3 Implementation details

#### 2.3.1 Scoring scheme

TWILIGHT determines sequence weights using the branch-proportional sequence weighting method (Thompson *et al.* 1994b), which gives higher weights to sequences with longer branches to prevent the alignment from being dominated by closely related sequences. If branch lengths are not provided, TWILIGHT assumes uniform branch lengths. For an alignment *A* with *N* sequences, the frequency $f_{A}$ of a character *c* at column *i* is computed using Equation (1). Here, $w_{A_{k}}$ represents the weight of the $k^{th}$ sequence, and *d* is a comparison function that returns 1 if $A_{i,k}$ equals *c* (match), and 0 otherwise (mismatch). These frequencies collectively form the profile of alignment *A*.


(1) $$f_{A_{i,c}}=\sum_{k}^{N}w_{A_{k}}d(A_{i,k},c),$$

The default substitution matrix in TWILIGHT is a slightly modified version of that used in MLAGAN (Brudno *et al.* 2003), +18 for match, +5 for transition, −8 for transversion, −50 for initial gap open penalty (*gop*), and −5 for gap extension penalty (*gep*) and mismatch penalty to gaps. When the position-specific gap penalty is enabled, gap penalties are adjusted using a simplified version of the approach used in ClustalW (Thompson *et al.* 1994a), as defined in Equation (2), where $gop_{A_{i}}$ represents the gap open penalty for column *i* in profile *A* and $w_{A}$ is the sum of sequence weights. TWILIGHT also accepts a user-defined substitution matrix using a command-line parameter --matrix (-x). Given a substitution matrix *s*, pairwise profile score ps(i,j) for column *i* in profile *A* and column *j* in profile *B* is calculated as Equation (3). For pairwise profile alignment, TWILIGHT applies Equation (4), which performs global alignment using pairwise profile scores and an affine gap penalty to determine the optimal alignment between two profiles.


(2) $$\begin{array}{c} gop_{A_{i}}=\{\begin{array}{c} \mathrm{gop}\times 0.3\times (1-\frac{f_{A_{i,-}}}{w_{A}}),f_{A_{i,-}}>0\\ \mathrm{gop},\mathrm{otherwise} \end{array}\\ gep_{A_{i}}=\{\begin{array}{c} 0.5\times \mathrm{gep},f_{A_{i,-}}>0\\ \mathrm{gep},\mathrm{otherwise} \end{array} \end{array}$$

(3) $$ps(i,j)=\frac{1}{w_{A}w_{B}}\sum_{p}^{\left\{A,C,G,T,N,-\right\}}\sum_{q}^{\left\{A,C,G,T,N,-\right\}}f_{A_{i,p}}f_{B_{j,q}}s(p,q)$$

(4) $$\begin{array}{cc} H(i,j)=\text{max}\{\begin{array}{c} H(i-1,j-1)+ps(i,j)\\ I(i-1,j-1)+ps(i,j)\\ D(i-1,j-1)+ps(i,j) \end{array} & \\ I(i,j)=\text{max}\{\begin{array}{c} H(i-1,j)+gop_{A_{i}}\\ I(i-1,j)+gep_{A_{i}} \end{array} & \\ D(i,j)=\text{max}\{\begin{array}{c} H(i,j-1)+gop_{B_{j}}\\ D(i,j-1)+gep_{B_{j}} \end{array} & \end{array}$$

#### 2.3.2 CPU parallelization techniques

TWILIGHT is implemented in C++ and leverages Threading Building Blocks (TBB) (Voss *et al.* 2019) to fully exploit thread-level parallelism available on multi-core platforms. TWILIGHT exploits inter-alignment parallelism of independent alignments determined by the scheduling algorithm described earlier, with each alignment processed in parallel on individual threads. Within each alignment, the calculation of profiles from alignment is also parallelized across multiple threads. Additionally, after aligning profiles, multiple sequences within each profile are updated in parallel. These multiple parallelism techniques ensure efficient utilization of computational resources throughout the progressive alignment process.

#### 2.3.3 GPU parallelization techniques

The GPU implementation of pairwise profile alignment in TWILIGHT is developed using CUDA and leverages three levels of parallelism, similar to the previous work on accelerating pairwise sequence alignment on GPU (Zeni *et al.* 2020). The first level is multi-GPU parallelism in which all alignments of the same order assigned by the scheduling algorithm are divided into multiple batches. Each batch contains at most as many alignments as the number of blocks in a single GPU (default: 2048). An atomic batch counter tracks the next batch to be processed. Once a GPU completes a batch, it fetches the next batch and increments the batch counter by 1. The second level of parallelism is inter-alignment parallelism, where each GPU thread block is assigned to perform a pairwise profile alignment (Fig. 1f). The third level of parallelism is intra-alignment parallelism, which exists at the wavefront (anti-diagonal) of the 2-D dynamic programming (DP) matrix of the Needleman–Wunsch algorithm modified for profile alignments. Different threads in a thread block compute multiple cells of a wavefront in parallel (Fig. 1f). By default, the block size is set to 256. If the wavefront is larger than the block size, there will be multiple rounds of execution per wavefront. To further accelerate the process, TWILIGHT uses recently introduced DPX instructions on NVIDIA GPUs (Elster and Haugdahl 2022) to compute the scores and pointers of the DP matrix. Additionally, to maximize the storage of traceback pointers in shared memory, TWILIGHT uses CUDA warp-level primitives to aggregate traceback pointers from adjacent threads, efficiently storing two pointers in a single byte.

#### 2.3.4 Memory-efficiency techniques

Multiple sequence alignment is a memory-intensive process and judicious usage of memory is key to scalability. TWILIGHT leverages the following strategies to enhance memory efficiency:

1. *Divide-and-conquer strategy and intermediate traceback path format for subalignment:* As discussed earlier (Section 2.2), the divide-and-conquer strategy in TWILIGHT controls memory usage by limiting the number of sequences processed simultaneously. However, this approach becomes more complex during the merging of two subalignments, which involves aligning a larger number of sequences. To address this challenge, TWILIGHT computes alignments using sequence profiles, whose sizes remain largely independent of the number of sequences. It then records the traceback path as a binary string, where a 1 indicates the insertion of a new gap and a 0 represents the insertion of an existing column. This compact representation enables the final alignment to be reconstructed and updated for each subalignment without loading all sequences into memory simultaneously.
2. *Banded alignment and tiling strategy:* To keep memory usage under control in the pairwise profile alignment step, TWILIGHT also uses a slightly modified version of the X-Drop algorithm (Zhang *et al.* 2000) to band the alignment. This ensures that memory usage scales linearly with the lengths of the sequences being aligned. However, this approach can still present challenges for GPU acceleration, as the on-chip shared memory, a limited resource, must be utilized for storing traceback pointers to maintain adequate performance. This issue was recently addressed by the TALCO tiling algorithm (Walia *et al.* 2024), which TWILIGHT incorporates (Fig. 1e). Briefly, TALCO operates in two phases: during the first phase, when the scoring wavefront remains within the tile boundary, indicated by a *marker* wavefront, traceback pointers for each cell are stored normally. In the second phase, instead of storing individual traceback pointers, TALCO maintains a convergence pointer that references a cell on the marker for only the current wavefront. The marker serves as the boundary between the two phases. If all convergence pointers point to the same cell—the convergence point—traceback begins from this point, and the next tile is processed upon completing the traceback. The method enables traceback pointers in a tile to be stored in constant memory, irrespective of the lengths of sequences being aligned, without affecting results. Because of limited shared memory on GPUs, the maximum anti-diagonal size on GPUs is limited to 1536, and the X-Drop value is set to 600 times larger than the gap-extension penalty, which corresponds to a value of 3000 in our default configuration. If the wavefront size exceeds this size or the alignment fails to complete within this X-Drop band, the alignment falls back to the CPU.

#### 2.3.5 Iterative mode

In progressive alignment, the quality of the MSA heavily depends on the accuracy of the guide tree. For scenarios in which a quality guide tree is unavailable, we use an iterative strategy, first introduced in MUSCLE (Edgar 2004) and later incorporated in several tools (Katoh *et al.* 2005, Liu *et al.* 2012, Mirarab *et al.* 2015). This strategy estimates an initial guide tree from input sequences and refines it over multiple iterations using the inferred MSA. To streamline this process, we provide a Snakemake workflow that integrates TWILIGHT’s core algorithm with various tree inference tools. By default, to maximize speed, PartTree (Katoh and Toh 2007) is used for estimating the initial guide tree from unaligned sequences and FastTree (Price *et al.* 2010) refines the guide tree based on the MSA. Other options include MashTree (Katz *et al.* 2019), MAFFT Tree (Katoh and Standley 2013) for initial guide trees, as well as RAxML (Stamatakis 2006), IQ-Tree (Minh *et al.* 2020) for subsequent iterations. Users are required to specify the desired number of iterations, up to a maximum of five, though empirically, three iterations are typically sufficient to achieve highly accurate results.

## 3 Experimental methodology

### 3.1 Datasets

We evaluated TWILIGHT and baseline tools using three simulated datasets and one real SARS-CoV-2 dataset. One alignment was simulated with RNASim (Guo *et al.* 2009) following the same methodology as Recursive MAGUS (Smirnov 2021), in which sequences were randomly sampled with counts ranging from 10 000 to 1 000 000 from a 1-million-sequence tree. The RNASim dataset has an average sequence length of approximately 1500 bases. Additionally, for alignments with varying sequence and branch lengths, we simulated two alignment datasets using AliSim (Ly-Trong *et al.* 2023) based on the general time reversible (GTR) model: one with long branches (average branch length of 0.01 substitutions/site) and the other with short branches (average branch length of 0.00001 substitutions/site). Both datasets were generated with an indel rate of 0.12 indels per substitution. The AliSim long-branched dataset consists of 10 000 sequences with an average length of 1000, while the AliSim short-branched dataset also contains 10 000 sequences but varies in length from 100 to 1 000 000. The true trees provided by RNASim and AliSim were used as the guide trees for the MSA tools. To construct the alignment of 8M SARS-CoV-2 sequences, we used the publicly available UShER phylogeny dated 8th September 2024, containing 8 112 719 tips (available at https://hgdownload.soe.ucsc.edu/goldenPath/wuhCor1/UShER_SARS-CoV-2/2024/09/08/), and downloaded the corresponding publicly available sequences from NCBI GenBank and COG-UK databases (Clark *et al.* 2016, Marjanovic *et al.* 2022).

### 3.2 Baseline tools

We used Clustal-Omega (Sievers *et al.* 2011), PASTA (Mirarab *et al.* 2015), MAFFT (Katoh and Standley 2013), Muscle5 (Edgar 2022), T-Coffee regressive mode (Garriga *et al.* 2019), and MAGUS recursive mode (Smirnov 2021) as baselines for comparisons with TWILIGHT. All tools, except MAFFT and Muscle5, were given the true tree from the simulator as the guide tree. PASTA and Clustal-Omega were restricted to a single iteration. We also excluded the runtime for the final tree estimation phase in PASTA for a fair comparison, as PASTA estimates a tree after constructing the MSA by default. All experiments, unless otherwise specified, were conducted using 32 cores on the parallelized version. See “Supplementary Materials” for detailed information on software versions and commands used.

### 3.3 Accuracy metrics

To evaluate the accuracy of alignments, we used FastSP 1.7.1 (Mirarab and Warnow 2011) to compute alignment errors by comparing the inferred alignments to the true simulated alignments. The FastSP error rate is determined as the average of the Sum-of-Pairs False Positive (SPFP) and False Negative (SPFN) rates provided by FastSP. For evaluating errors in tree inference, we used MAPLE 0.6.12 (De Maio *et al.* 2023) to compute the normalized Robinson-Foulds distance (nRF) (Robinson and Foulds 1981) of the inferred tree to the true tree from the simulator.

### 3.4 Hardware and execution environment

TWILIGHT CPU version and all baseline tools were executed on a CPU instance, which features nodes with 2 AMD EPYC 7V13 64-core processors and 512 GB of memory. TWILIGHT GPU version was executed on a separate GPU instance, which consists of 48 Intel^®^ Xeon^®^ Gold 5220R CPU cores and 2 NVIDIA RTX A6000 GPUs, along with 376 GB of memory. Notably, the GPU instance is equipped with less powerful CPU cores than the CPU instance, as this is typically the case in a cloud infrastructure.

## 4 Results

### 4.1 TWILIGHT outperforms state-of-the-art MSA tools in both speed and accuracy on challenging alignments

**Figure 2.**
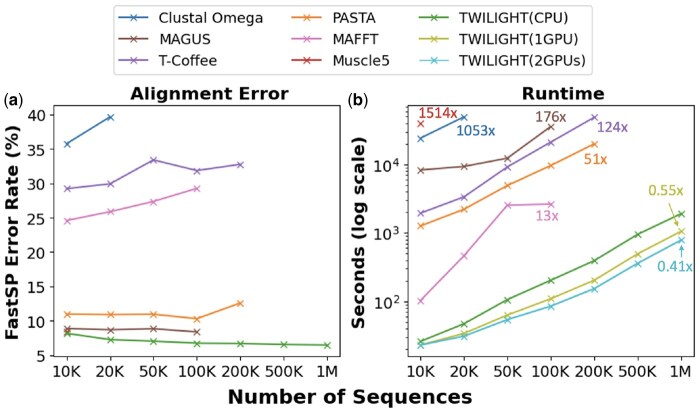
Accuracy and scalability with sequence count. (a) Accuracy and (b) Speed comparison of TWILIGHT with other MSA tools. We provide the ratio of the runtimes of different tools relative to TWILIGHT CPU version on the largest dataset they could handle.

We compared the speed and accuracy of TWILIGHT to state-of-the-art MSA tools using the RNASim dataset (see Section 3). This offers a challenging benchmark and has been widely used in previous studies (Mirarab *et al.* 2015, Nguyen *et al.* 2015, Smirnov and Warnow 2021). TWILIGHT stood out to be the most accurate as well as the fastest MSA tool among all tested tools (Fig. 2). At 10 000 sequences, only MAGUS and Muscle5 achieved comparable accuracy to TWILIGHT, having a FastSP error rate of around 8%. However, Muscle5 did not scale to more sequences and MAGUS failed to complete the alignment of 200 000 sequences within the time limit (24 h). PASTA’s error rate was about 3% higher and all other tools had significantly higher error rates. TWILIGHT also showed improvement in error rate as the number of sequences being aligned increased, reaching 6.48% with 1 million sequences, whereas the error rate either remained unchanged (MAGUS) or increased with the number of sequences for other tools. Figure 2a shows the error rate of only the CPU version of TWILIGHT, as the GPU version was within 0.1% margin, with small differences arising from the different rounding modes in the two versions.

In terms of runtime, TWILIGHT CPU version outperformed other CPU-based tools, achieving a 13-fold speedup over the closest competitor, MAFFT, for 100 000 sequences (Fig. 2b), and between 47-fold and 176-fold compared to the remaining tools. The TWILIGHT GPU version delivered additional speedups of 1.8- and 2.4-fold speedups of the CPU version with 1 and 2 GPUs, respectively (Fig. 2b). Since the CPU version used more powerful cores, the speedups for the GPU version over the CPU version were higher, 4.5- and 6.1-fold, respectively, when we executed the CPU version on GPU instances.

TWILIGHT’s handling of gappy columns (Section 2.2) not only benefited speed and accuracy but also produced the most compact MSAs. When disabling the removal of gappy columns, TWILIGHT produced alignment of 10 000 sequences 12 times slower and with a 8.8% higher FastSP error rate. For 100 000 sequences, TWILIGHT’s MSA size was 1.6 GB, close to the true alignment size of 1.4 GB, but MAFFT, T-Coffee, MAGUS, and PASTA produced MSAs of sizes 1.9 GB, 2.2 GB, 21.9 GB, and 18 GB, respectively. The significantly larger file sizes for PASTA and MAGUS can be attributed to the transitivity merger and the graph clustering merger strategies, respectively. The difference was even more dramatic at 200 000 sequences, where the true alignment size was 3.3 GB: TWILIGHT’s MSA remained efficient at 3.6 GB, whereas PASTA’s MSA exploded to 61.5 GB. TWILIGHT CPU (1 GPU) could align a million sequences in only 32 min (18 min)–all other tools either crashed or failed to complete the alignment within 24 h, confirming that TWILIGHT can perform ultrafast tall alignments.

### 4.2 TWILIGHT scales linearly to sequence length and efficiently handles available parallelism

**Figure 3.**
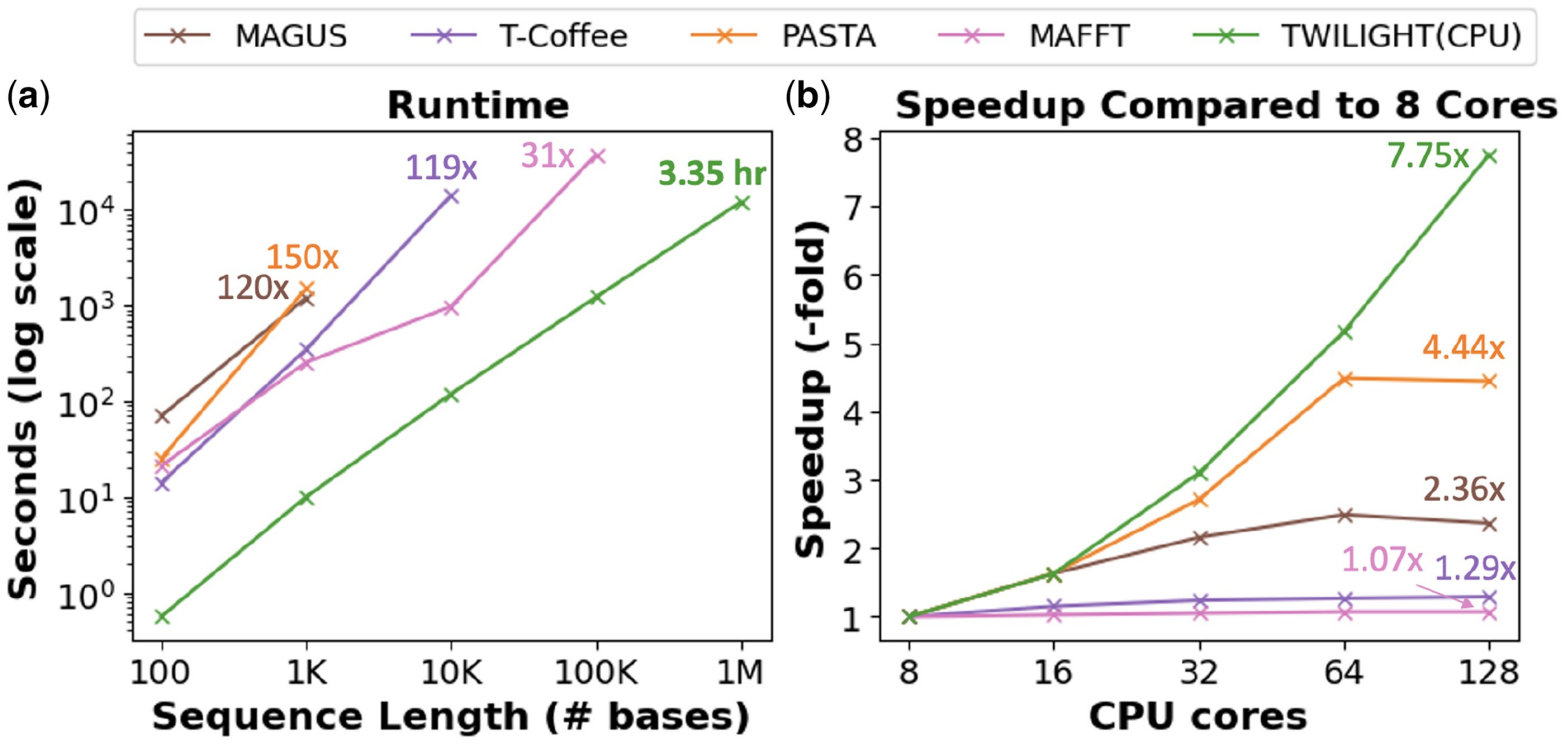
Comparison of scalability of TWILIGHT with respect to (a) sequence length and (b) available CPU parallelism against the four fastest MSA tools from the previous experiment. We presented the runtime ratios of various tools relative to TWILIGHT on the longest dataset each tool could handle, as well as the speedup achieved when scaling from 8 to 128 CPU cores.

We used AliSim short-branched sequences for testing scalability on long sequences. Figure 3a showed that TWILIGHT scales linearly to sequence length, and is the only tool that could complete the alignment of 10 000 sequences with 1 million bases in length within 24 h. PASTA crashed at 10 000-base alignment because of java.lang.OutOfMemoryError when running external tools, while the scaling of other tools was limited by long runtimes. Through its tiling strategy, TWILIGHT can handle even longer sequences, up to tens of millions of bases (see “Supplementary Materials”). This capability opens the possibility of aligning chromosome-scale sequences, provided they have not undergone nonlinear rearrangements such as duplications, translocations, or inversions.

TWILIGHT’s implementation also efficiently utilizes available parallelism. TWILIGHT achieved a 7.75-fold speedup when the number of cores was scaled by 16-fold, from 8 to 128 cores. T-Coffee and MAFFT provided barely any speedups with more cores, whereas the speedups of PASTA and MAGUS saturated at 64 cores, achieving 4.48-fold and 2.49-fold speedups, respectively.

### 4.3 TWILIGHT’s iterative mode generates accurate alignments in the absence of guide trees

We evaluated the performance of TWILIGHT’s iterative mode on the AliSim long-branched dataset. We used two different configurations for this mode–one using the MAFFT Tree (Katoh and Standley 2013) as the initial guide tree for the first iteration and the other using PartTree (Katoh and Toh 2007). FastTree (Price *et al.* 2010) was used for the subsequent iterations. We compared TWILIGHT to MAGUS and PASTA because they are the most competitive in terms of speed and accuracy. PASTA estimated trees with FastTree, which is its default option. Since MAGUS does not estimate a tree post-alignment, we also applied FastTree to MAGUS’s alignment for evaluating tree quality. To ensure fairness of comparison, this experiment only used 8 CPU cores because HMMER (Eddy 2009, Finn *et al.* 2011) in PASTA was unable to create more than 8 threads on the CPU instance.

**Figure 4.**
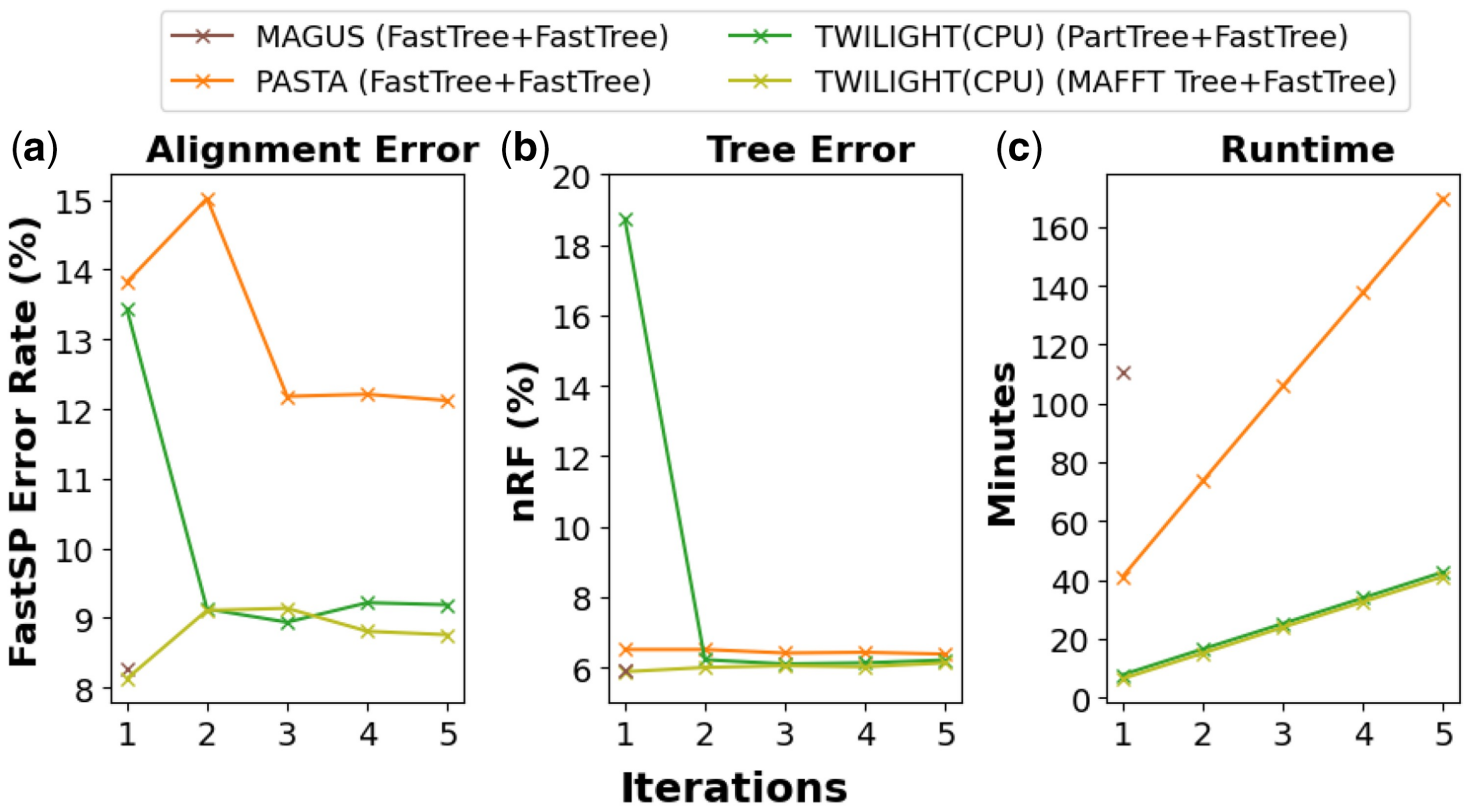
Performance comparison of TWILIGHT iterative mode with PASTA and MAGUS in terms of (a) alignment error rate, (b) tree error rate, and (c) runtime. Legends indicate the guide tree estimation tool used for first and subsequent iterations for all tools.

Both TWILIGHT configurations achieved comparable alignment and tree error rates to MAGUS after the second iteration and outperformed PASTA in alignment accuracy (Fig. 4a). Despite PartTree producing a low-quality guide tree in the first iteration, TWILIGHT successfully refined both the alignment and the tree over subsequent iterations. Considering the runtime for three iterations (default in TWILIGHT iterative mode), TWILIGHT demonstrated a 4.4-fold speedup compared to PASTA and a 4.6-fold speedup compared to MAGUS, with slightly better final tree (Fig. 4c). We also observed that TWILIGHT alignment only accounted for 7% of the total runtime–the remaining 93% of the runtime was spent in the tree inference step. Our future work will involve accelerating this primary bottleneck. Additionally, our results (Table 1) show that even when the initial guide tree quality is poor, e.g. when the nRF of the input tree is 40%–50% compared to the true tree, TWILIGHT can still achieve highly accurate alignments (within 14% error, which is comparable to PASTA), suggesting that it is not highly sensitive to the quality of the input guide tree.

**Table 1.** Performance comparison of TWILIGHT using different guide trees.^a^

	RNASim		AliSim long-branched	
	Tree error (%)	Alignment error (%)	Tree error (%)	Alignment error (%)
True Tree	0	7.83	0	8.41
MAFFT Tree	42.2	8.68	16.9	8.13
PartTree	86.5	11.58	51.2	13.42

a We used the MAFFT tree and PartTree to generate guide trees and computed the nRF to the true (simulated) tree.

### 4.4 TWILIGHT can adapt to platforms with limited memory constraints

**Figure 5.**
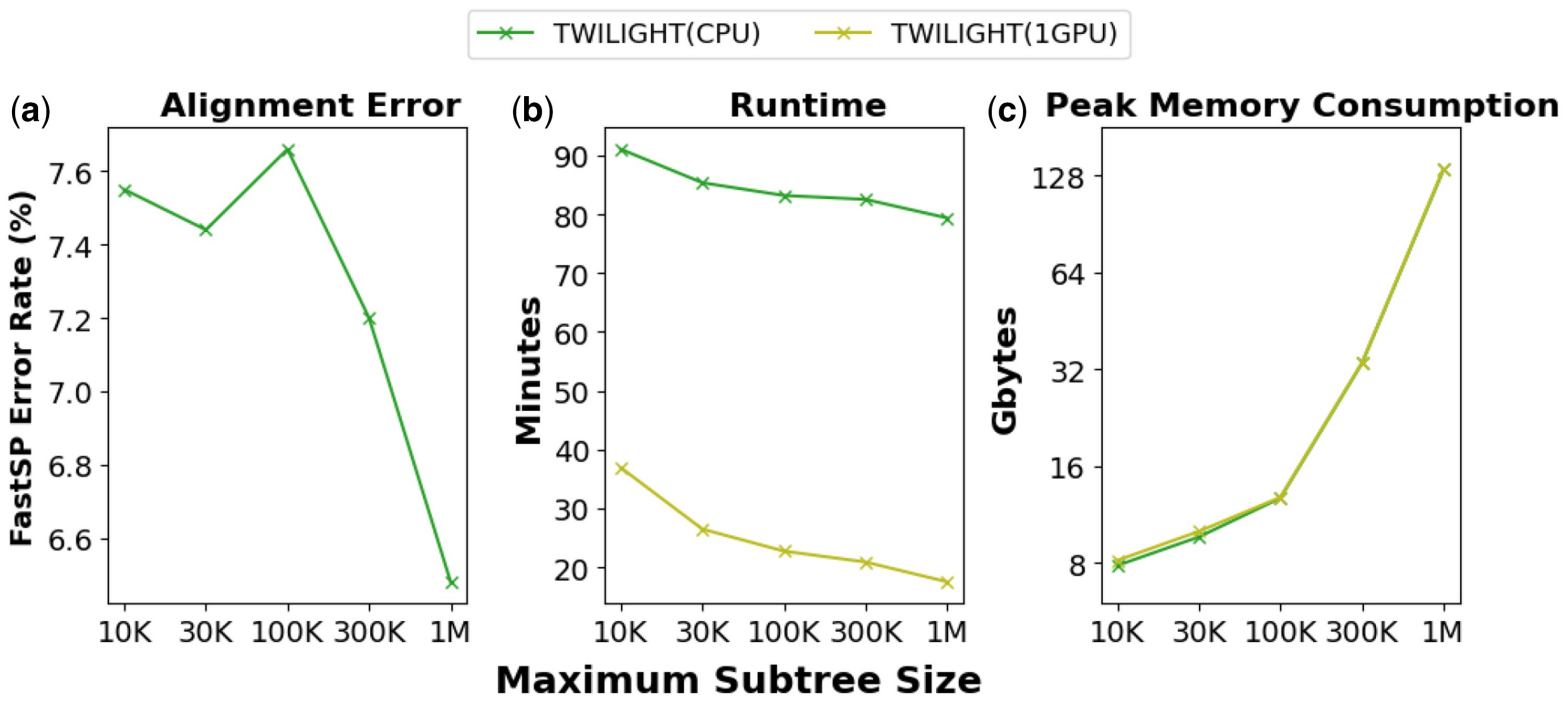
(a) Accuracy, (b) memory efficiency and (c) runtime analysis for different subtree sizes in the CPU and GPU versions of TWILIGHT using the 1-million-sequence RNASim dataset. Both TWILIGHT CPU and GPU versions were executed on the GPU instance.

TWILIGHT incorporates several innovative strategies that empower it to handle ultralarge alignments, even on memory-constrained platforms like laptops and personal servers (see Section 2.3.4). We evaluated TWILIGHT’s adaptability to memory-limited platforms on the 1-million-sequence RNASim dataset (Fig. 5). Without the divide-and-conquer strategy (dividing into subtrees, see Section 2.3.4), both CPU and GPU versions of TWILIGHT required 133 GB of memory. By setting the maximum subtree size to 100 000, the peak memory usage of both versions was reduced 10-fold to just 13 GB, making it possible to run an ultralarge alignment of over a million sequences on typical laptops and personal computers. A smaller subtree size, however, can lead to fewer independent alignments, reducing parallelism. Additionally, overheads such as writing subalignments and repeatedly reading the unaligned sequence file contribute to slightly longer runtimes in this mode. This effect is more obvious in the GPU version, which was 2.1-fold slower when the maximum subtree size was set to 10 000, compared to only a 1.15-fold slowdown on the CPU version (Fig. 5b). It is also worth noting that after dividing the initial tree into subtrees, the progressive alignment for merging may not strictly follow the original guide tree topology, potentially resulting in a minor accuracy loss (Fig. 5a). Despite these trade-offs, TWILIGHT uniquely empowers users to perform ultralarge alignments, which were previously impossible on memory-constrained devices, while maintaining excellent runtime efficiency and accuracy. We also compared the peak memory consumption of TWILIGHT and other tools using the 100 000-sequence RNASim dataset, as detailed in the “Supplementary Materials.” TWILIGHT’s divide-and-conquer strategy enabled it to use 7–16× less memory compared to other tools, while maintaining high accuracy.

### 4.5 TWILIGHT produces ultralarge MSA of 8M SARS-CoV-2 genomes in only 28 h

We demonstrate TWILIGHT’s ability to align large volumes of long sequences on real-world data by constructing MSA of 8 112 719 SARS-CoV-2 sequences. Besides being tall, this alignment is also wide, as each sequence is roughly 30 000 bases in length. So far, large structural mutations have not been observed in SARS-CoV-2, so a linear MSA can serve as a valid whole-genome alignment of all sequences. For performing this alignment, we used the UShER tree dated 8th September 2024, curated and shared by UCSC (McBroome *et al.* 2021, Turakhia *et al.* 2021), as the guide tree for TWILIGHT. Remarkably, TWILIGHT completed the alignment in just 28 h on our GPU instance, making it, to the best of our knowledge, the first tool to produce an MSA of all publicly available SARS-CoV-2 sequences.

Although there was a need to produce an alignment of this scale during the COVID-19 pandemic, previous MSA tools failed to handle this scale. While a few heuristic approaches (Moshiri 2021, Tang *et al.* 2022, Chen *et al.* 2023) were developed to speed up the process by performing pairwise alignment with respect to a reference sequence, their outputs cannot be classified as true MSAs since they either discarded the insertions found relative to the reference sequence or left them unaligned.

To assess the accuracy of TWILIGHT MSA, we manually checked whether it contained indels of high significance reported in prior studies. Specifically, we focused on the sequences descending from the major variants of concern (VoC)—Omicron sub-lineages BA.1, BA.2, and BA.3, Delta (B.1.617.2), Gamma (P.1), and Alpha (B.1.1.7)—and compared their insertions and deletions (indels) with the 22 lineage-defining indels described in a previous study (Hill *et al.* 2022, Li *et al.* 2023). We found that 21 of 22 reported lineage-defining indels were present in all sequences belonging to the corresponding VoCs. The only exception was a deletion (NC_045512.2: 22 029–22 034), reported by (Hill *et al.* 2022, Li *et al.* 2023) to have occurred in B.1.1.7, but it too was found in a subset (24.1%) of 631 341 B.1.1.7 descendants in the MSA. Overall, the consistent presence and distribution of significant indels throughout the alignment indicate that the TWILIGHT MSA is of high quality and is unlikely to have major accuracy issues. We have also made this MSA available (https://zenodo.org/records/14722035) for the benefit of broad researchers.

## 5 Conclusion and future work

We introduced TWILIGHT, a MSA tool designed to overcome the scalability limitations of existing solutions. TWILIGHT includes GPU support and is *uniquely* capable of performing alignments that are simultaneously *tall* (many sequences) and *wide* (long sequences). Our experiments highlight its significant advantages over state-of-the-art MSA tools, delivering massive speedups with comparable or even better accuracy than the most accurate alternatives. By incorporating innovative parallel computing techniques and memory-efficient strategies, TWILIGHT can efficiently process ultralarge datasets, even on memory-constrained devices, making it broadly accessible to researchers. TWILIGHT is also the first tool to have produced an MSA of over 8 million SARS-CoV-2 genome sequences.

With the continued exponential growth of genomic data, TWILIGHT is poised to be a transformative tool in bioinformatics, enabling detailed insights from large-scale genomic datasets. Future enhancements to TWILIGHT could include incorporating more sensitive methods for detecting highly divergent alignments (remote homology), handling local alignments, and accelerating the guide tree construction step, which is currently the main bottleneck in the iterative mode. We also aim to expand TWILIGHT into a multiple whole-genome aligner capable of handling nonlinear genomic rearrangements, such as inversions, translocations, and duplications.

## Supplementary Material

btaf212_Supplementary_Data
