# Supplementary material for: Ultrafast and ultralarge multiple sequence alignments using TWILIGHT
Source: Bioinformatics. 2025 Jul 15;41(Suppl 1):i332–41. doi: 10.1093/bioinformatics/btaf212 (PMC12261412; doi:10.1093/bioinformatics/btaf212)
Supplement: btaf212_Supplementary_Data [file btaf212_supplementary_data.pdf]

Supplementary Materials

Software Versions and Commands Used

Tool	Version	Command
**TWILIGHT**	**0.1.0**	twilight --cpu 32 -i <sequences> -t <tree> -o <output> --psgop y
**PASTA**	**1.9.2**	run_pasta.py -d <rna/dna> --iter-limit=1 -i <sequences> -t <tree> --num-cpus 32 -o <output>
**T-Coffee**	**13.46.1.b8b01e06**	t_coffee -reg -thread=32 -seq <sequences> -tree <tree> -outfile <output>
**MAGUS**	**0.2.0**	magus -np 32 -i <sequences> -t <tree> -o <output> --recurse true
**MAFFT**	**7.525**	mafft --auto --ep 0.123 --quiet --thread 32 --anysymbol <sequences> > <output>
**Clustal-Omega**	**1.2.4**	clustalo --threads 32 -i <sequences> --guidetree-in <tree> --iter 1 -o <output>
**Muscle5**	**5.3.linux64**	muscle5 -super5 <sequences> -output <output> -threads 32

Table 1: Versions and commands used for TWILIGHT and baseline tools.

Additional Experimental Results

AliSim Short-branched Simulated Sequences		
Sequence Count	100	100
Sequence Length	5,000,000	50,000,000
Runtime (seconds)	4790	53548
Peak Memory Usage (Gbytes)	28.498	256.429

This experiment was conducted using 32 Intel® Xeon® Silver 4216 CPUs

None of the baseline tools were able to complete these tasks within the 24-hour limit.

Table 2: Runtime and peak memory usage of TWILIGHT on chromosome-scale sequences.

100,000-sequence RNASim Dataset							
Tools	TWILIGHT			PASTA	T-Coffee	MAGUS	MAFFT
`--max-subtree`	10000	30000	∞ (default)	N/A	N/A	N/A	N/A
Peak Memory	0.836	2.310	10.462	11.942	13.985	11.436	6.516
Error Rate (%)	8.00	7.54	6.75	10.31	31.86	8.39	29.27

Unit of peak memory usage: Gbytes

Table 3: TWILIGHT's peak memory consumption compared to other tools. The `--max-subtree` option in TWILIGHT allows lowering main memory requirements to a fraction of other tools while maintaining high accuracy.

RNASim Datasets								
	Unit (%)	10000	20000	50000	100000	200000	500000	1000000
TWILIGHT	SPFN	8.16	7.24	7.01	6.71	6.66	6.50	6.43
	SPFP	8.19	7.30	7.07	6.79	6.76	6.59	6.52
	TC	0.62	0.93	0.47	0.53	0.65	0.69	0.53
	Expansion	123	124	118	113	108	103	99
PASTA	SPFN	10.94	11.10	11.13	10.46	12.88		
	SPFP	11.03	10.71	10.78	10.15	12.29		
	TC	2.20	2.86	3.07	4.67	5.78		
	Expansion	303	477	827	1255	1862		
T-Coffee	SPFN	39.42	40.68	44.89	42.10	44.75		
	SPFP	19.31	19.15	21.90	21.61	20.78		
	TC	0.33	0.44	0.26	0.27	0.17		
	Expansion	113	128	138	158	174		
MAGUS	SPFN	9.40	9.09	9.14	8.96			
	SPFP	8.38	8.32	8.56	7.82			
	TC	3.09	3.68	3.80	5.02			
	Expansion	460	699	1064	1528			
MAFFT	SPFN	29.97	32.00	34.39	36.69			
	SPFP	19.22	19.75	20.33	21.84			
	TC	0.51	0.34	0.31	0.48			
	Expansion	112	117	123	129			
Clustal Omega	SPFN	48.77	54.81					
	SPFP	22.72	24.52					
	TC	0.19	0.26					
	Expansion	57	60					
Muscle5	SPFN	8.39						
	SPFP	8.65						
	TC	0.67						
	Expansion	103						

SPFN: 1 - number of correctly aligned pairs / total number of aligned pairs in the true alignment

SPFP: 1 - number of correctly aligned pairs / total number of aligned pairs in the estimated alignment

TC: number of correctly aligned columns / total number of aligned columns in the true alignment.

Expansion: number of columns in the estimated alignment / number of columns in the true alignment.

Table 4: Detailed evaluation metrics for the experiment on the RNASim dataset.
